# Enhanced synthesis of 5-hydroxy-l-tryptophan through tetrahydropterin regeneration

**DOI:** 10.1186/2191-0855-3-70

**Published:** 2013-12-09

**Authors:** Ryotaro Hara, Kuniki Kino

**Affiliations:** 1 Research Institute for Science and Engineering, Waseda University, 3-4-1 Ohkubo, Shinjuku-ku, Tokyo 169-8555, Japan; 2 Department of Applied Chemistry, Faculty of Science and Engineering, Waseda University, 3-4-1 Ohkubo, Shinjuku-ku, Tokyo 169-8555, Japan

**Keywords:** 5-hydroxy-l-tryptophan, l-phenylalanine 4-hydroxylase, Tetrahydropterin, NAD(P)H, Cofactor regeneration

## Abstract

5-Hydroxy-l-tryptophan (5-HTP) is a naturally occurring aromatic amino acid present in the seeds of the African plant *Griffonia simplicifolia*. Although 5-HTP has therapeutic effects in various symptoms, efficient method of producing 5-HTP has not been established. In this study, we developed a novel cofactor regeneration process to achieve enhanced synthesis of 5-HTP by using modified l-phenylalanine 4-hydroxylase of *Chromobacterium violaceum*. For the synthesis of 5-HTP using *Escherichia coli* whole cell bioconversion, l-tryptophan and 5-HTP degradation by *E. coli* endogenous catabolic enzymes should be considered. The tryptophanase gene was disrupted using the λ red recombination system, since tryptophanase is postulated as an initial enzyme for the degradation of l-tryptophan and 5-HTP in *E. coli*. For regeneration of the cofactor pterin, we screened and investigated several key enzymes, including dihydropteridine reductase from *E. coli*, glucose dehydrogenase from *Bacillus subtilis*, and pterin-4α-carbinolamine dehydratase from *Pseudomonas syringae*. Genes encoding these three enzymes were overexpressed in an *E. coli* tryptophanase-deficient host, resulting in the synthesis of 0.74 mM 5-HTP in the presence of 0.1 mM pterin and the synthesis of 0.07 mM 5-HTP in the absence of regeneration of pterin. These results clearly indicated the successful regeneration of pterin. Following optimization of the reaction conditions, 2.5 mM 5-HTP was synthesized with cofactor regeneration, while 0.8 mM 5-HTP was recovered without cofactor regeneration under the same reaction conditions, suggesting that the principle described here provides a new method for cofactor regeneration.

## Introduction

5-Hydroxy-l-tryptophan (5-HTP) is a serotonin pathway metabolite of l-tryptophan in the brain. 5-HTP controls the concentration of serotonin, and therefore plays a significant role in the serotonin pathway. In this pathway, l-tryptophan is hydroxylated by tryptophan 5-hydroxylase to form 5-HTP, following which aromatic amino acid decarboxylase (Zhu and Juorio 1995) catalyzes the formation of 5-hydroxytryptamine (serotonin). The tryptophan hydroxylation reaction has been identified as the rate-limiting step in the serotonin pathway, and this reaction regulates the formation of serotonin. Hence, serotonin levels are elevated by oral administration of 5-HTP, leading to therapeutic effects in the case of various symptoms, including depression (Angst et al. 1977; Persson and Roos 1967; Takahashi et al. 1975; van Praag and de Hann 1980), chronic headache (Bono et al. 1984; Ribeiro 2000), and insomnia (Guilleminault et al. 1973; Wyatt et al. 1971). Moreover, 5-HTP acts as an antioxidant agent (Cadenas et al. 1989; Lysek et al. 2003).

Numerous studies have reported the drug evaluation and medicinal properties of 5-HTP; however, the production of 5-HTP is currently dependent mainly on extraction from the seeds of the African plant *Griffonia simplicifolia*. This method is not suitable for efficient production of 5-HTP; therefore, a more practical method providing better yield is required. Alternatively, pterin-dependent aromatic amino acid hydroxylases (Fitzpatrick 2000), which are family of enzymes that require tetrahydropterin as a cofactor, including phenylalanine hydroxylase, and both tyrosine and tryptophan hydroxylases (rate-limiting enzymes for catecholamine and serotonin pathways, respectively), have been identified as candidates capable of efficiently synthesizing hydroxy aromatic amino acids. These enzymes are widely distributed throughout vertebrate species, and the human pterin-dependent aromatic amino acid hydroxylases in particular have been well elucidated. Tryptophan 5-hydroxylase is capable of efficiently synthesizing 5-HTP via the hydroxylation of l-tryptophan; however, this method is not suitable for industrial use due to low hydroxylation activity and poor stability when expressed both in prokaryotes and eukaryotes (Wang et al. 2002). In contrast, some prokaryotes, such as *Chromobacterium violaceum* (Nakata et al. 1979; Onishi et al. 1991) and *Pseudomonas aeruginosa* (Zhao et al. 1994), also produce l-phenylalanine 4-hydroxylase, but tryptophan 5-hydroxylase is not found in any of these microbes.

l-Phenylalanine 4-hydroxylase from *C. violaceum* (CviPAH) can hydroxylate the C-5 position of l-tryptophan as well as the C-4 position of l-phenylalanine (Fujisawa and Nakata 1987; Nakata et al. 1979; Pember et al. 1987). However, the hydroxylation rate of l-tryptophan is relatively lower than that of l-phenylalanine (~0.4%). In our previous study, we performed structure-based rational designing of CviPAH, and the resulting mutant enzyme, CviPAH-L101Y-W180F, exhibited a 5.2-fold higher *k*_cat_ value than the wild-type enzyme during l-tryptophan hydroxylation (Kino et al. 2009). This enzyme reaction should have high potential for practical production of 5-HTP because no by-products are formed, and the substrate, l-tryptophan, is available through industrial microbial fermentation performed using metabolically engineered *Corynebacterium glutamicum* (58 g/L) (Ikeda and Katsumata 1999) and *Escherichia coli* (54.6 g/L) (Azuma et al. 1993), both of which provide excellent yield. However, CviPAH-L101Y-W180F still requires pterin as a cofactor for enzyme activity, which is similar to the requirements of other types of aromatic amino acid hydroxylases. We attempted to regenerate the cofactor to overcome this problem, since pterin is an expensive compound. Here, we report the development of an enhanced l-tryptophan hydroxylation process coupled with regeneration of the cofactors pterin and NAD(P)H. Pterin biosynthesis and regeneration are observed in mammals; therefore, we cloned and reconstructed the corresponding enzymes derived from various microorganisms in *E. coli* with the aim of developing an enhanced enzymatic selective synthesis method for the production of 5-HTP.

## Materials and methods

### Chemicals and strains

6,7-Dimethyl-5,6,7,8-tetrahydropterine (DMPH_4_) hydrochloride was purchased from Sigma (St. Louis, MO). All other chemicals were of the highest analytical grade available. *Bacillus subtilis* ATCC 23857 and genomic DNA of *Pseudomonas syringae* ATCC BAA-978D were obtained from American Type Culture Collection.

### Disruption of the chromosomal *tnaA* gene in *E. coli*

*E. coli* BL21(DE3) *tnaA*^*–*^ was constructed using the λ Red recombination system (Datsenko and Wanner 2000). A deletion cassette for *tnaA* gene, which encodes tryptophanase, was amplified by PCR using FRT-PKG-gb2-neo-FRT (Gene Bridges; Heidelberg, Germany) as template DNA and the following primers: forward 1 (5′-TGTAATATTCACAGGGATCACTGTAATTAAAATAAATGAAGGATTATGTAAATTAACCCTCACTAAAGGGCG-3′) and reverse 1 (5′-TGTAGGGTAAGAGAGTGGCTAACATCCTTATAGCCACTCTGTAGTATTAATAATACGACTCACTATAGGGCTC-3′). KOD -plus- DNA polymerase (Toyobo; Osaka, Japan) was used, and the PCR program was as follows: 94°C for 2 min and 25 cycles each of 94°C for 20 s, 60°C for 15 s, and 68°C for 2 min. According to the manufacturer’s recommendation, mutant strains were selected and transferred onto Luria Bertani agar (10 g/L Bacto Tryptone, 5 g/L Bacto Yeast Extract, 10 g/L NaCl and 15 g/L Bacto Agar) containing 30 μg/mL kanamycin, and the resulting *tnaA*-deficient mutant was collected and used as a host for the subsequent experiments. Degradation of l-tryptophan and 5-HTP was performed in a reaction mixture containing 100 mM HEPES-NaOH buffer (pH 7.5), 5 mM l-tryptophan, 0.1 mM DMPH_4_, 0.1 mM FeSO_4_, 50 mM d-glucose, 1% (v/v) Triton X-100, and *E. coli* whole cells (OD_660_ = 50) in a total volume of 1 mL.

### Construction of expression vectors

The gene encoding PAH-L101Y-W180F was amplified by PCR using the following primers: forward 2 (5′-ATATA*CATATG*AACGACCGCGCCGACTTTGTGGTGC-3′; the *Nde* I site is underlined) and reverse 2 (5′-TTCAT*CTCGAG*ATTAGACGTCTTCGGTATCCGCCCATCC-3′; the *Xho* I site is underlined) from pEPAH-L101Y-W180F (Kino et al. 2009). The gene encoding pterin-4α-carbinolamine dehydratase (PCD, GenBank Protein ID: AAZ35428) was amplified using the following primers: forward 3 (5′-AATAG*CCATGGG*TACCTTGAATCAAGCCCATTGCG-3′; the *Nco* I site is underlined) and reverse 3 (5′-AATAT*GTCGAC*ATTATTTGCGGCCTTCGGCGCCACTG-3′; the *Sal* I site is underlined) from the chromosomal DNA of *P. syringae*. The amplified genes were inserted into the corresponding restriction site of pETDuet-1 (Novagen; Madison, Wl). The resulting plasmid was labeled pEDPCD-PAH-L101Y-W180F and used for the expression of PCD and PAH-L101Y-W180F genes in *E. coli*. The gene encoding dihydropteridine reductase (DPR, GenBank Protein ID: AAC73679) was amplified by PCR using the following primers: forward 4 (5′-TCATTAT*CATATG*GATATCATTTCTGTCGCCTTAAAGCGT-3′; the *Nde* I site is underlined) and reverse 4 (5′- TGTAATAG*CTCGAG*TTTACACTTCGGTTAAGGTGATGTTTTGCG-3′; the *Xho* I site is underlined) from the chromosomal DNA of *E. coli* K-12 MG1655. The glucose dehydrogenase (GDH, GenBank Protein ID: CAB12201) gene was amplified using the following primers: forward 5 (5′-CTATA*GAATTC*GTATCCGGATTTAAAAGGAAAAG-3′; the *Eco*R I site is underlined) and reverse 5 (5′-*ATTATGTCGACA*TTAACCGCGGCCTGCCTGGAATGAAG-3′; the *Sal* I site is underlined) from the chromosomal DNA of *B. subtilis*. The amplified genes were inserted into the corresponding restriction site of pACYCDuet-1 (Novagen). The resulting plasmid was labeled pAGDH-DPR and was used for the expression of GDH and DPR genes in *E. coli*.

### Preparation of recombinant *E. coli* BL21(DE3) *tnaA*^–^ expressing PAH-L101Y-W180F, PCD, DPR and GDH genes

*E. coli* BL21(DE3) *tnaA*^–^ harboring pEDPCD-PAH-L101Y-W180F and pAGDH-DPR was cultivated at 37°C in LB medium containing 50 μg/mL ampicillin and 30 μg/mL chloramphenicol. Isopropyl-β-d-thiogalactopyranoside (IPTG) was added to the culture when the OD_660_ reached 0.5, to a final concentration of 0.1 mM, and the cells were then cultivated at 25°C with shaking. After a 20-h induction period, *E. coli* BL21(DE3) harboring plasmids were harvested by centrifugation at 5,000 × *g* for 5 min at 4°C. The cell pellet was washed twice with 200 mM HEPES-NaOH buffer (pH 8.0) and resuspended in the same buffer. To confirm the expression of enzymes, SDS-PAGE analysis was carried out on supernatant of disrupted cells.

### Synthesis of 5-HTP thorough cofactor regeneration

5-HTP was synthesized in a reaction mixture containing 100 mM HEPES-NaOH buffer (pH 8.0), 5 mM l-tryptophan, DMPH_4_ at different concentrations, 0.1 mM FeSO_4_, 50 mM d-glucose, 1% (v/v) Triton X-100, and *E. coli* whole cells (OD_660_ = 50) in a total volume of 1 mL. The reaction was performed at 30°C. After the reaction, cells were removed by centrifugation at 20,000 × *g* for 5 min at 4°C. The concentration of 5-HTP in the reaction mixture was determined by high performance liquid chromatography as described previously (Kino et al. 2009).

## Results

### Construction of the *E. coli* BL21(DE3) *tnaA*^–^ strain

**Figure 1:**
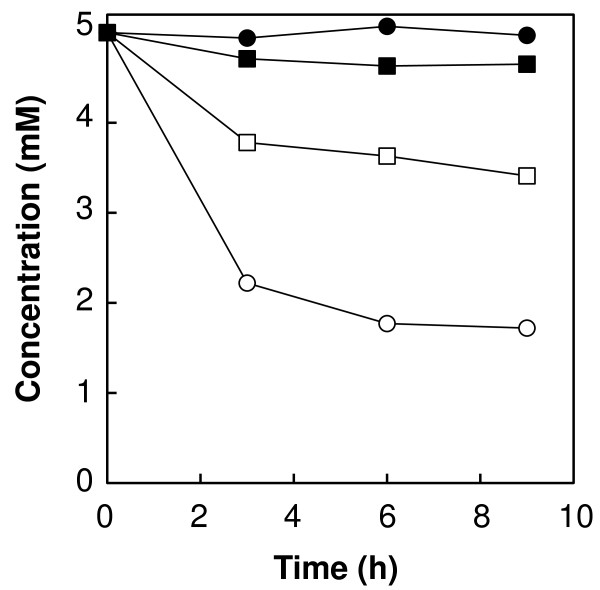
**Degradation of l-tryptophan and 5-HTP using *E. coli *cells.***Open circles*, l-tryptophan using wild-type *E. coli*; *filled circles*, l-tryptophan using *E. coli tnaA* disruptant; *open squares*, 5-HTP using wild-type *E. coli*; *filled squares*, 5-HTP using *E. coli tnaA* disruptant.

During the production of 5-HTP, the endogenous metabolic enzymes of *E. coli* (e.g., tryptophanase) degrade l-tryptophan to indole, pyruvate, and ammonia; and 5-HTP is degraded to 5-hydroxyindole, pyruvate, and ammonia (Nakazawa et al. 1972a; Nakazawa et al. 1972b; Watanabe and Snell 1972). We also confirmed the degradation of 5-HTP as well as of l-tryptophan in the wild-type *E. coli* whole cell reaction (Figure 1), and then attempted to delete the gene encoding tryptophanase (*tnaA*), which is the enzyme that initially degrades l-tryptophan, to overcome these drawbacks.

To prevent the degradation of l-tryptophan and 5-HTP, the *tnaA* gene of *E. coli* BL21 (DE3) was disrupted using the Red/ET recombination system. Consequently, the degradation of l-tryptophan and 5-HTP was clearly inhibited by disruption of the *tnaA* gene of *E. coli* (Figure 1), suggesting that this host strain contributes to enhance 5-HTP production.

### Synthesis of 5-HTP catalyzed by PAH without DMPH_4_ regeneration

To achieve l-tryptophan hydroxylation using PAH-L101Y-W180F, it is necessary to add the coenzyme DMPH_4_ to the reaction mixture. Using *E. coli* BL21(DE3) *tnaA*^*–*^ whole cells expressing only PAH-L101Y-W180F gene, 5-HTP was synthesized in the presence of 0.1–5 mM DMPH_4_ and was observed to increase with increase in the concentration of DMPH_4_ up to 18% (0.9 mM) at 5 mM DMPH_4_, indicating that synthesis of 5-HTP is coenzyme DMPH_4_-dependent. We also attempted to develop higher yield synthesis of 5-HTP by reduced addition of DMPH_4_.

### Effect of coexpression of the DPR, GDH, and PCD genes

**Figure 2:**
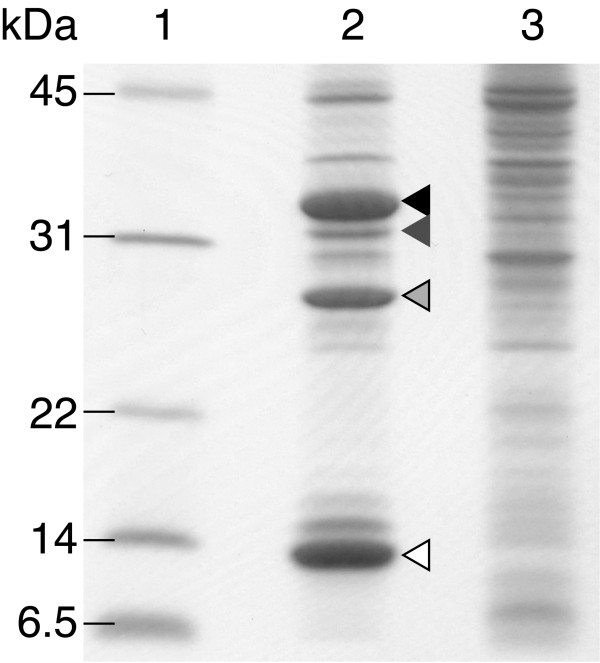
**SDS-PAGE analysis of soluble proteins in *E. coli *cells.** Lanes 1, molecular mass standards with size indicated; 2, soluble protein of *E. coli* expressing heterologous genes; 3, soluble protein of *E. coli* (negative control). Triangles indicate as follows: *filled*, l-phenylalanine 4-hydroxylase; *dark gray*, glucose dehydrogenase; *light gray*, dihydropteridine reductase; *open*, pterin-4α-carbinolamine dehydratase.

**Figure 3:**
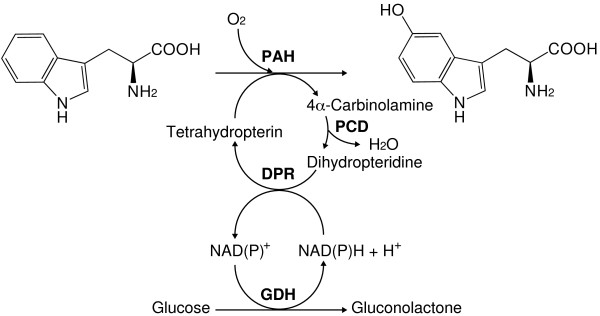
**Scheme of 5-HTP synthesis through cofactor regeneration.** PAH, l-phenylalanine 4-hydroxylase; PCD, pterin-4α-carbinolamine dehydratase; DPR, dihydropteridine reductase; GDH, glucose dehydrogenase.

To construct DMPH_4_ regeneration through oxidation-reduction reactions, two plasmids were constructed: pEDPCD-PAH-L101Y-W180F and pAGDH-DPR. Both plasmids were introduced into *E. coli* BL21(DE3) *tnaA*^–^, and the genes encoding PAH-L101Y-W180F, PCD, DPR, and GDH were coexpressed in the same *E. coli* cells. SDS-PAGE analysis revealed that all of enzymes were successfully expressed in *E. coli* compared to negative control (Figure 2). Hence, we applied this strain as a catalyst for l-tryptophan hydroxylation using a whole cell reaction (Figure 3).

**Figure 4:**
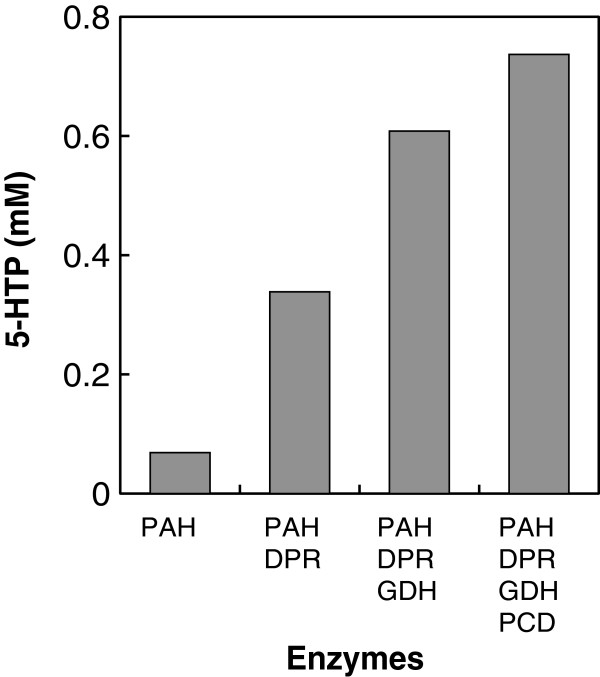
**Effects of DMPH**_**4 **_**regeneration enzymes on synthetic yield of 5-HTP using PAH.** The basal reaction condition is described in the text. In this experiment, 0.1 mM DMPH_4_ was applied.

In order to initially regenerate DMPH_4_ from 6,7-dimethyl-5,6,7,8-dihydropterine (DMPH_2_), which is an oxidized form of DMPH_4_, DPR from *E. coli* K-12 (Vasudevan et al. 1988) and PAH-L101Y-W180F were coexpressed in the same *E. coli* cells. DPR was selected for DMPH_4_ regeneration because it exhibits tetrahydropterin-dependent NAD(P)H oxidoreductase activities. This approach resulted in an increase in 5-HTP synthesis compared to that obtained with PAH-L101Y-W180F alone with 0.1 mM DMPH_4_ (Figure 4). Furthermore, 0.35 mM 5-HTP was synthesized in the presence of DPR with only 0.1 mM DMPH_4_, suggesting that DPR served as a DMPH_4_ regeneration enzyme.

To further accelerate DMPH_4_ regeneration using *E. coli* endogenous NAD(P)H, it is necessary to regenerate NAD(P)H from NAD(P)^+^ in the *E. coli* cell. For NAD(P)H regeneration, GDH from *B. subtilis* was selected since it serves as a cofactor in NAD(P)H regeneration. Moreover, GDH has high specific activity and prefers NADP^+^ over NAD^+^ (Yun et al. 2005). Similarly, DPR prefers NADPH over NADH. Thus, the genes encoding GDH, DPR, and PAH-L101Y-W180F were coexpressed in *E. coli* BL21(DE3) *tnaA*^–^. The coexpression of the GDH gene, in addition to the PAH-L101Y-W180F and DPR genes in *E. coli*, led to a 1.6-fold increase in 5-HTP as compared to that for the PAH-L101Y-W180F and DPR genes alone, suggesting that NAD(P)H regeneration stimulated DMPH_4_ regeneration.

During human pterin regeneration, aromatic amino acid hydroxylation is followed by the conversion of pterin to pterine-4α-carbinolamine. Pterine-4α-carbinolamine is subsequently converted to dihydropteridine, and DPR catalyzes the reduction of dihydropteridine to tetrahydropterin. In this process, PCD catalyzes the dehydration of pterine-4α-carbinolamine to produce dihydropteridine. However, pterine-4α-carbinolamine is affected by a nonenzymatic rearrangement with opening of the pyridine ring, yielding the 7-substituted pterin (Davis et al. 1991; Schallreuter et al. 1994). Additionally, in the absence of PCD, 7-substituted pterin is formed and competes with the natural cofactor tetrahydropterin for PAH, leading to a critically reduced hydroxylation rate (Davis et al. 1991). Therefore, to accelerate DMPH_4_ regeneration and increase the 5-HTP yield, more PCD was added and the corresponding gene was coexpressed in *E. coli* BL21(DE3) *tnaA*^–^. The results of this approach indicated a nearly 11-fold (0.74 mM) increase in the synthesis yield of 5-HTP when compared to samples lacking cofactor regeneration (Figure 4).

### Optimization of reaction conditions for synthesis of 5-HTP

Enhanced synthesis of 5-HTP was investigated using PAH-L101Y-W180F, and an oxidation-reduction enzyme process for DMPH_4_ regeneration was established. It was then necessary to optimize the reaction conditions using *E. coli* whole cells with the four enzymes coexpressed in the same *E. coli* cell.

**Figure 5:**
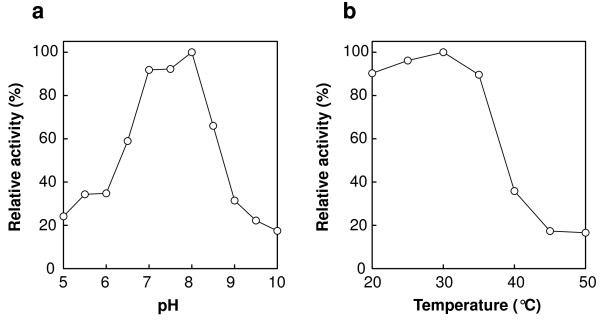
**Optimal pH and temperature for the synthesis of 5-HTP, using the *E. coli *whole cell reaction. (a)** The reaction conditions, except for pH and buffer, are described in the text. The following buffers were used: acetate buffer, pH 5.0–5.5; maleate buffer, pH 6.0; MOPS-NaOH buffer, pH 6.5; HEPES-NaOH buffer, pH 7.0–8.0; Tris–HCl buffer, pH 8.5; borate-NaOH buffer, pH 9.0–10. **(b)** The reaction conditions, except for temperature, are described in the text.

Effects of pH and temperature on 5-HTP synthesis were examined over a range of pH values (pH 5–10) and temperatures (20–50°C). The maximal yield of 5-HTP was obtained at pH 8.0 and 30°C (Figure 5).

**Figure 6:**
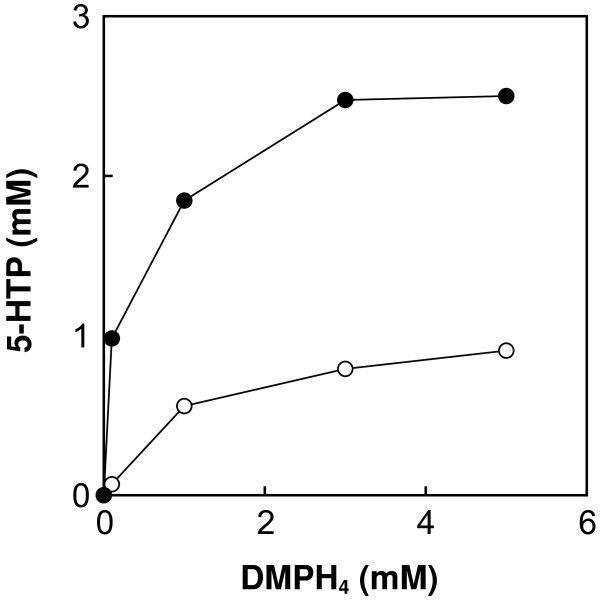
**Effect of DMPH**_**4 **_**concentration on the synthesis of 5-HTP, using the *E. coli *whole cell reaction.***Open circles*, l-tryptophan hydroxylation using *E. coli tnaA* disruptant expressing PAH gene alone; *filled circles*, l-tryptophan hydroxylation using *E. coli tnaA* disruptant expressing the genes encoding PAH, PCD, DPR, and GDH.

Finally, the effects of DMPH_4_ concentration (0.1–5 mM) on 5-HTP synthesis were determined at optimal pH and temperature. The molar yield of 5-HTP reached 50% when DMPH_4_ exceeded 3 mM (Figure 6).

## Discussion

In the present study, we developed a novel cofactor regeneration system for l-tryptophan hydroxylation to synthesize 5-HTP. In this coupling system of pterin and NAD(P)H regeneration, 5-HTP was synthesized in quantities exceeding the concentration of the pterin added, which is necessary for l-tryptophan hydroxylation.

We recently used site-directed mutagenesis to engineer CviPAH, which exhibited high l-tryptophan hydroxylation activity. Comparison of the crystal structures of CviPAH with human l-tryptophan 5-hydroxylase led to the hypothesis that L101 and W180 in CviPAH were responsible for cofactor and substrate affinity, respectively, and we consequently engineered the CviPAH L101Y-W180F mutant enzyme (Kino et al. 2009). While higher hydroxylation activity toward l-tryptophan was achieved by CviPAH L101Y-W180F, the cofactor pterin is still essential for the aromatic amino acid hydroxylase reaction, and because pterin is an expensive reagent for industrial applications, there is a demand for a more cost-effective process to provide pterin. To accomplish this, we focused on developing a pterin-regeneration system. Additionally, since human pterin recycling enzymes from the brain and other tissues have been investigated and it is known that NAD(P)H is necessary for pterin regeneration, we hypothesized that if this process could be reconstructed in *E. coli*, l-tryptophan hydroxylation with regeneration of the cofactors pterin and NAD(P)H could be established, leading to efficient 5-HTP synthesis (Figure 3). NAD(P)H can be regenerated using dehydrogenase-coupling processes, including GDH, formate dehydrogenase, and alcohol dehydrogenase; therefore, we utilized GDH from *Bacillus subtilis* to regenerate NAD(P)H and pterin due to its high enzyme activity and stability.

Currently, 5-HTP is produced by extraction from the seeds of the African plant *G. simplicifolia*. Several attempts have been made to synthesize 5-HTP using chemical or enzymatic syntheses; however, these have proven to be unsuitable from an industrial perspective. Therefore, we focused our attention on microbial l-phenylalanine 4-hydroxylase because this enzyme hydroxylates l-tryptophan to 5-HTP, and l-phenylalanine to l-tyrosine. Although engineered CviPAH exhibited high l-tryptophan hydroxylation activity, the enzyme required l-tryptophan and equimolar concentrations of the cofactor pterin, which is an expensive and scarce compound in the industry. Hence, we proposed and constructed a novel cofactor regeneration process coupled with amino acid hydroxylation. Using microbial genome information, we reconstituted three enzymes, PCD, DPR and GDH, for pterin regeneration, and coexpressed the corresponding genes in *E. coli*. When the resulting *E. coli* tryptophanase-deficient mutant expressing CviPAH-L101Y-W180F, PCD, DPR, and GDH was used as a whole cell catalyst, higher l-tryptophan hydroxylation activity was observed than that seen with *E. coli* expressing the gene encoding CviPAH alone (Figure 4), suggesting that pterin regeneration was functionally affected.

Indeed, the regeneration process is effective for the production of 5-HTP to some extent, although this approach could not effect conversion of all of the available l-tryptophan into 5-HTP. As shown in Figure 4, the maximum yield of 5-HTP was 0.74 mM in the presence of 0.1 mM DMPH_4_, suggesting that the total turnover number is 7.4 mole/mole. On the other hand, the total turnover number is less than 1 mole/mole in case of >3 mM DMPH_4_ addition (Figure 6). Previous studies suggested that GDH should promote further NAD(P)H regeneration; however, complete pterin regeneration was still difficult to achieve, which may be due to instability of the pterin. Several studies have reported that pterin-4α-carbinolamine, an oxidized form of pterin, can spontaneously convert to an undesirable 7-substituted pterin (Curtius et al. 1990a; Curtius et al. 1990b; Davis et al. 1991; Thony et al. 2000). Once this 7-substituted compound is formed, the pterin regeneration cycle is hampered, since the hydroxylase reaction is partially uncoupled (Schallreuter et al. 1994). To avoid the formation of 7-substituted pterin and accelerate pterin regeneration, PCD, or “phenylalanine hydroxylase-stimulating protein” (Hauer et al. 1993; Huang et al. 1973), was supplied to stimulate the regeneration system. While a minor stimulatory effect was observed, only a slight increase in 5-HTP was achieved (Figure 4). This effect is likely due to the unnecessary accumulation of pterin analog such as 7-substituted pterin, and this problem must be resolved in order to achieve complete conversion of l-tryptophan to 5-HTP.

In summary, we report the development of a novel pterin regeneration process for enhanced l-tryptophan hydroxylation. Although the efficiency of pterin regeneration is expected to improve with further enhancements to the regeneration enzymes, this regeneration process represents a powerful tool for practical amino acid hydroxylation using aromatic amino acid hydroxylases, including l-phenylalanine 4-hydroxylase, l-tyrosine 3-hydroxylase, and l-tryptophan 5-hydroxylase.

## Competing interests

The authors declare that they have no competing interests.
